# Construction, observation and knowledge abstraction for go endgames on small boards

**DOI:** 10.1038/s41598-024-57338-x

**Published:** 2024-03-22

**Authors:** Chia-Ming Hsu, Hung-Cheng Lin, Yueh-Ting Chen, Chih-Wen Hsueh, Tsan-sheng Hsu

**Affiliations:** 1 Institute of Information Science, Academia Sinica, Taipei, 115201 Taiwan; 2 Department of Computer Science and Information Engineering, National Taiwan University, Taipei, 106216 Taiwan

**Keywords:** Computer science, Scientific data

## Abstract

A Go endgame database consists of optimal game values and moves for every legal arrangement of no more than *S* pieces on an *N* by *N* board. This paper describes methods for constructing such databases when $1 < N \le 5$ and $S = N^2$. When cycles of plies with lengths greater than 4 are encountered, two rules, one allowing cycles and the other disallowing them, are implemented. Observations and knowledge are obtained for these endgames, which may elucidate the fundamental properties of the popular game Go. First, the optimal game values are different when *N* is even and odd, regardless of whether the repetition of positions is allowed. When *N* is odd, the first player can occupy the whole board, while this is not the case when *N* is even. Second, allowing cycles makes the first and second players equal in strength when *N* is even, whereas the first player always dominates when *N* is odd. Using the state-of-the-art open-source deep learning Go engine KataGo to correctly solve a given position as an indicator, factors affecting level of difficulty are found, including the distributions of the optimal game values among all legal plies and the cardinality and values of the true optimal plies. A simple formula is designed that works on more than 10% of the positions so that positions with a given level of difficulty can be found with a high probability.

## Introduction

Go is an ancient board game that is popular internationally^1–4^. Over the years, people have played Go with various board sizes, ranging from 4^5^ to 19. Different rules are used for scoring^6,7^; these rules include the setting of a **Komi** value^8^, which represents the compensation given to the second player due to the advantage of the first player obtained from the initiative, and different treatments are used when a repetition of plies occurs^9^. Regarding this last point, cycles of plies or repetition of positions, whose lengths are always even, may occur. To encourage meaningful play, Go forbids the formation of length-2 cycles, called **Ko**, which can be created easily by players. Different Go rules involve different complicated treatments for allowing and disallowing other types of cycles^10^.

Regardless of which variant is used, they are all enjoyable, though it is not known why. In this research, through the construction of Go **endgame databases**^11^, which consist of optimal game values and moves for every legal arrangement of no more than *S* pieces on an *N* by *N* board, we aim to shed light on the above questions. Our approach relies on constructed endgame databases instead of developing **solvers**^12^ to find optimal plies when particular board positions are given. Although a solver can quickly find solutions for a particular board position, and thus can be used to determine the optimal ply on initially empty boards, this does not give an aggregated picture of solutions for all possible positions. It will take too much time to run the solver on all possible positions. On the other hand, the construction of an endgame database usually requires much more time but gives all the results at once. However, the amortized time needed to solve a position is much shorter than the time needed for a solver to complete this task.

We use the classical retrograde analysis algorithm^13^ to construct the databases. Due to the large size of the databases and the need to address cycles in plies, we develop memory-efficient methods tailored for handling graphs with cycles^14^ that cannot fit into the main memory. After the databases are constructed, we perform data analysis to obtain an overall picture of the results. We find the following interesting properties. First, the optimal game values are different when *N* is even and odd, regardless of whether repeated positions are allowed. When *N* is odd, the first player can occupy the whole board, while this is not the case when *N* is even. Second, allowing cycles makes the first and second players equal in strength when *N* is even, whereas the first player is always dominant when *N* is odd.

Furthermore, taking the time required by the state-of-the-art open-source deep learning Go engine KataGo to correctly solve a given position as an indicator, the factors affecting the level of difficulty are found to include the distributions of the optimal game values among all the legal plies and the cardinality and values of the true optimal plies. A simple formula is designed so that positions with a given level of difficulty can be found with a high probability.

The remainder of this paper is organized as follows. In Sect. "Preliminaries", we present preliminaries regarding Go rules and related work. In Sect. "Methodologies", we describe our main methods, including the algorithms and data structures used. In Sect. "Experimental results", we describe the experimental settings and results. In Sect. "Discussion", we analyze the databases and discuss interesting points found in the analysis. In Sect. "Concluding remarks and future work", we present the conclusion and possible future work.

## Preliminaries

### Rules of Go

**Figure 1:**
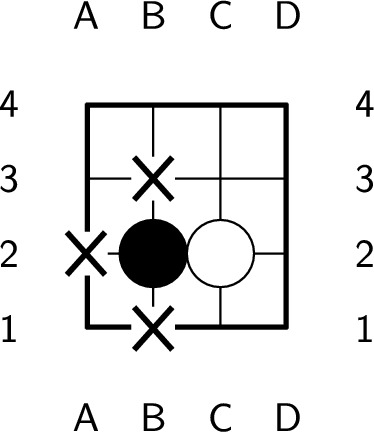
A black stone with 3 liberties, labeled with ’X’.

**Figure 2:**
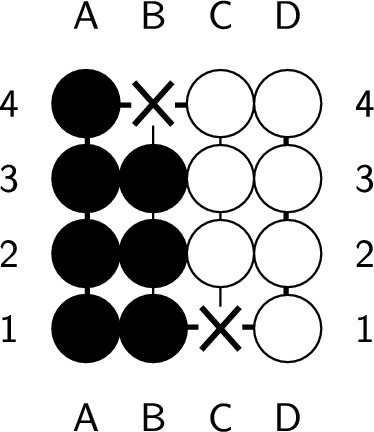
An example of seki. In the figure, ’X’ represents a shared liberty.

**Figure 3:**
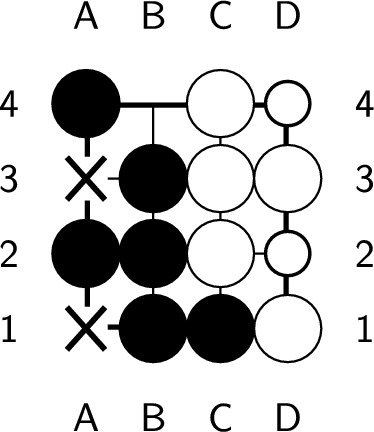
An example of enclosed empty intersections. In the figure, ’X’ and ’O’ represent empty intersections enclosed by the black and white players, respectively.

Each turn in the game of Go involves one of two actions: playing a stone or passing. These actions are referred to as a ply, and the game ends when both players pass consecutively. Stones in Go can be placed only at intersections. Each empty intersection connected to a stone is considered a liberty of the stone. The liberties of the black stone in Fig. 1 are labeled. According to the Go rules, any connected or single stone must have at least one liberty. Otherwise, the stones are considered dead and must be removed from the board. To balance the advantages of both players, komi is introduced as a bonus score for the second player. Additionally, seki is a special pattern in which neither player benefits from playing a stone, as shown in Fig. 2. Liberties that are shared by both players in a seki are called shared liberties, as labeled in Fig. 2. Moreover, it is important to count the enclosed empty intersections when calculating the score, as demonstrated in Fig. 3.

#### Scoring: territory/area

The number of occupied intersections is used in Go to determine the winner, which is the player who scores more points after deducting the komi from the first player. There are two major ways to count, namely, area scoring and territory scoring^6,7^. Before calculating the score, both scoring methods require the removal of dead stones. In area scoring, both the stones and the empty intersections enclosed by the stones are considered to be the occupied intersections. In addition, the shared liberties in seki are equally divided between both players. If the number of shared liberties, that is, the empty intersections that have both black and white stones as neighbors, in seki is odd, then the last liberty is given to the player who passed first. In contrast, in territory scoring, only the intersections enclosed by stones of the same color are counted, not stones. Thus, shared liberties in seki are not counted at all. The final score is calculated by subtracting the number of stones captured in the play.

#### Cycles: Ko and others

In Go, if there are stones that have no liberties, the stones are removed. Since stones can be captured, it is possible to cause cycles. Cycles can be divided into many types according to length, where a 2-length cycle is called *ko* and occurs frequently during play. To keep the game going, 2-length cycles are forbidden. However, cycles of length other than 2 are handled differently under different rules. Some Go rules allow such cycles, and the game results in a draw, but some Go rules forbid any kind of cycle.

#### Major variations among rules

Currently, there are many Go rules, such as the AGA Rules, Chinese Rules, Ing Rules, New Zealand Rules, Japanese Rules, and Korean Rules. Area scoring is adopted by the Chinese Rules, Ing Rules, AGA Rules, and New Zealand Rules, then territory scoring is adopted by the Japanese Rules and Korean Rules. According to the AGA Rules and New Zealand Rules, all cycles are forbidden. However, according to the type of cycle, the game may end in a draw; cycles are forbidden in the Chinese Rules. According to the Japanese Rules and Korean Rules, games are judged as having no result and may be replayed after a cycle occurs. Ing Rules are similar to Chinese Rules but use more complex rules for dealing with cycles. Table 1 shows a comparison of Go rules^15^.

**Table 1:** Comparison of rules for $19\times 19$ Go.

	Scoring method	No suicide rule	Repeated positions	Komi
AGA rules	Area	Yes	Forbidden	7.5
Chinese rules	Area	Yes	Forbidden or a draw	7.5
Ing rules	Area	No	Depends on ko rules	8
Japanese rules	Territory	Yes	Game ends with no result	6.5
Korean rules	Territory	Yes	Game ends with no result	6.5
New Zealand rules	Area	No	Forbidden	7

### Related work

We first describe previous Go endgame databases. In 2001, Bouzy Bruno used retrograde analysis to construct $1\times 1$ Go, $2\times 2$ Go, and $3\times 3$ Go endgame databases^16^. In his paper, the repetition of states is forbidden. In the $1\times 1$ Go scenario, the game ends in a draw because both players cannot play anywhere. In addition, the black player can win 1 in $2\times 2$ Go due to the repetition rule. In $3\times 3$ Go, if the black player plays at the center, then the white stones cannot avoid being captured. Therefore, the black player can win 9 in $3\times 3$ Go. We next describe the Go solver results.

Erik C.D. Van Der Werf et al. used a search-based approach to solve Go problems on small boards^17^. They wrote a program, Migos, to solve Go boards of sizes up to $5\times 5$. Migos is based on alpha-beta and implements a transposition table, symmetry lookup, internal unconditional bounds, and move ordering. They found Go solutions for four different ko rules: basic, Japanese, approximate SSK, and SSK. According to the SSK ko rules, the black player can win 2 and 25 in $4\times 4$ Go and $5\times 5$ Go, respectively.

The Go program Crazy Stone, using Monte Carlo tree search (MCTS), won $9\times 9$ Go in the 11th Computer Olympiad^18^. Afterward, it was found that MCTS is more suitable for Go. Cheng-Wei Chou et al.^19^ used Meta-MCTS to solve $7\times 7$ Go in 2011. Although his algorithm does not completely solve $7\times 7$ Go, it provides strong opening books. In addition to MCTS, machine learning (ML) has been a good method for Go in recent years. KataGo was created by David J. Wu and is a Go program based on AlphaGo Zero and AlphaZero^20^. In 2021, the $7\times 7$ Go opening books calculated by KataGo were uploaded^21^. Although there is no proof that $7\times 7$ Go has been solved, the authors believe it is very close to being solved.

Yang, Bohong et al. published an approach to train Go models without prior knowledge of komi^22^. Moreover, the Go model can learn komi during training. Their model uses Tromp Taylor rules and estimates that the komi is 9, 2, and 4 for $3\times 3$ Go, $4\times 4$ Go, and $6\times 6$ Go, respectively. The best first moves for $3\times 3$ Go, $4\times 4$ Go, and $6\times 6$ Go are b2, c3, and d4, respectively. The authors also provided the solutions of their model for $6\times 6$ Go, $6\times 7$ Go, and $7\times 8$ Go.

## Methodologies

In this paper, we construct Go endgame databases using AGA Rules^23^ with two variations. One treats cycles of length $\ge$ 4 as draws and the other does not allow any cycles. In accordance with the AGA Rules, area scoring is used for score calculation. Additionally, cycles and suicides are not allowed, which means that any moves that cause a cycle or a suicide are considered illegal moves. Finally, we set the komi to 0.

This chapter is divided into two parts. The first part describes the algorithms for building the endgame databases and addressing cycles. In the second part, we focus on the data structures and the approach used to reduce memory usage and the amount of storage space needed.

### Retrograde algorithm

Retrograde analysis is a widely used algorithm in the construction of endgame databases^13^. Initially, all states are labeled as unknown, except for the states where the game outcomes are already known, which are the leaves of the game tree. Retrograde analysis involves calculating the endgame value of each state by backtracking from the leaves of the game tree. There are various ways to implement retrograde analysis algorithms, including retrograde analysis algorithms with external memory^24^ and parallel retrograde analysis algorithms^25^.

#### Repeated forward checking with no cycles

We use the forward-checking variant to implement the retrograde analysis algorithm, which means that we check each state repeatedly and calculate the endgame value of the state based on the endgame values of its children. Since cycling causes the endgame values of the states in the cycles to continue changing, this makes it impossible to stop retrograde analysis. When there is no child that can win and there is a child with an unknown endgame value, to prevent the effect of cycles on retrograde analysis, the endgame value is not updated. Otherwise, we will change the endgame value of the current state to the best endgame value for the current player based on the children.

#### Loop handling—SCC

In Go, there are various kinds of cycles. The reason a cycle occurs is that there is no way for any state in a cycle to win. After performing retrograde analysis, there are some states whose endgame values are unknown; these states are called *unstable states*. The other states are called *stable states*. The unstable states can be divided into two types, in-cycle and out-cycle states, according to whether the state is in a cycle. The states in cycles are called *in-cycle states*, and the others are called *out-cycle states*. To prevent cycles in Go, we need to determine the in-cycle states in the game tree and calculate the endgame values when removing the edges that can cause a cycle to form. After finding the endgame values of in-cycle states, we calculate the endgame values of out-cycle states.

Each in-cycle state is reachable from any other in-cycle state in the same cycle, which is a strongly connected component (SCC)^14^. A cycle can be composed in a very complicated way in a graph. In addition to the single 4-cycle, two 4-cycles can form a larger cycle. Moreover, an out-cycle state is also an SCC with only one state. According to the types of the states in the SCCs, we divide the SCCs into in-cycle SCCs and out-cycle SCCs.

After finding the in-cycle and out-cycle states, we first calculate the endgame values for each in-cycle SCC by depth-first search (DFS) to iterate through all the states in an in-cycle SCC and remove the edges that cause cycles. For those states whose edges are deleted, the endgame values can be calculated using other children. However, because whether an edge causes a cycle is related to the visiting path, the endgame value of the same state may be different when the visiting path is different. Therefore, we have to calculate the endgame values for a state when the visiting paths are different.

After calculating the states in the SCCs, we calculate the endgame values of all the out-cycle SCCs. Because each out-cycle SCC consists of only one vertex, we use retrograde analysis on all out-cycle SCCs to calculate the endgame values. In addition, some in-cycle SCCs are connected to other in-cycle SCCs through out-cycle SCCs, so we calculate the in-cycle SCCs again. We repeat the calculation of the endgame values of the in-cycle SCCs and the out-cycle SCCs until the endgame values of all SCCs have been calculated. Although we have already found the endgame values for each in-cycle and out-cycle state, the task is not over. If we update the endgame values of the SCCs, then the stable states should also be updated because the stable states may find a better endgame value in the SCCs. Moreover, after updating the stable states, unstable states also may find better endgame values. Therefore, we need to update both the unstable and stable states until no state can be changed.

#### Implementations

$N \le 4$: Since we use the forward-checking variant to implement the retrograde analysis algorithm, it is necessary to know the number of children for each state to calculate the endgame value of each state. However, the overhead of calculating the children of each state is very high. Therefore, we store the children of each state first and use them directly. While performing retrograde analysis, we load the children and endgame values of each state and perform retrograde analysis until all the states are stable. Finally, we address the cycles, and the endgame database is completed. To increase the speed, we also use parallel computing to calculate the endgame values of several states simultaneously.

$N = 5$: In $5\times 5$ Go, the process of building the endgame databases is similar to that for $4\times 4$ Go. However, as the number of states increases, the states cannot be completely loaded into the memory. Therefore, we divide all the states into several groups. We perform retrograde analysis for one group at a time, so we only need to load the children of those states into the group, which significantly reduces the memory requirements.

On the other hand, since the SCC sizes of $5\times 5$ Go also increase, we use alpha-beta search instead of DFS to calculate the endgame values to save time.

### Implementation of endgame databases

In Go, each possible configuration is referred to as a *state*, which comprises a board position, a board status, and a turn. The board position indicates the positions of all stones, and we encode board positions as numbers using board serials. The board status pertains to the status of the board position, such as ko or pass. The board status is represented by a numerical code known as the board status code. The turn records who plays next. To generate all the legal states in Go, we enumerate all the board serials and board statuses and verify their legality. For more information on the data structures and illegal conditions, please refer to Supplementary Information SI. Table 2 displays the numbers of legal and illegal states, and Table 3 presents the quantity of stored children.

For each size of Go board, we build separate endgame databases instead of extending smaller ones. This is because additional rows and columns prevent some stones from being captured, resulting in different winning strategies and game results. Additionally, it is important to note that only the states in which it is the black player’s turn need to be saved since the game value of a state remains the same after changing the current player and reversing the colors of all the stones.

**Table 2:** Numbers of legal and illegal states.

Size	Legal states	Illegal states
$2\times 2$	157	996
$3\times 3$	38,651	420,102
$4\times 4$	76,046,601	1,333,239,544
$5\times 5$	163,665,274,870	26,102,281,617,863

**Table 3:** Numbers of children stored.

Size	Numbers of children stored
$2\times 2$	260
$3\times 3$	109,944
$4\times 4$	345,910,100
$5\times 5$	565,815,779,101

**Figure 4:**
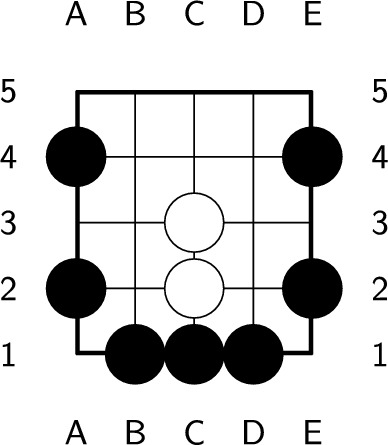
Isomorphic board (a).

**Figure 5:**
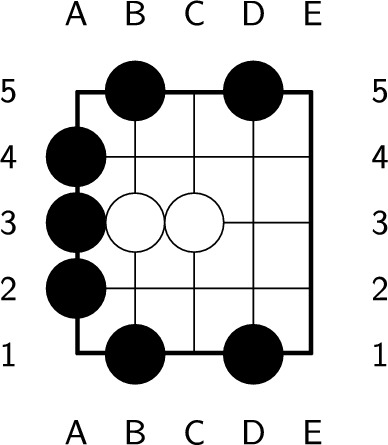
Isomorphic board (b).

$N \le 4$: Since the maximum number of board serials is $3^{16} = 43,046,721$, which can be stored in 26 bits ($\lceil \log _2{3^{16}} \rceil = 26$), we put the board serials in bit 1 to bit 26. The maximum number of status codes is $10101_2$, which can be stored in 5 bits, namely, bit 27 to bit 31. Finally, the total number of bits in the state index is 31. Thus, we use a 32-bit integer to store a state index. Equation (1) shows the formula that converts a board serial and a status code into a state index in $4\times 4$ Go. We traverse through all the state indices and check whether they are legal. However, some of these states are redundant because the board serial is larger than $3^{16}$; thus, the states are marked as illegal. Once the legal states are obtained, we calculate all the children of these legal states and use the CSR format to store them.

1 $$\begin{aligned} Index_{B} = Status_{B} \times 2^{26} + Serial_{B} \end{aligned}$$

$$N = 5$$:

Since the number of board serials in $5\times 5$ Go is enormous, we use isomorphic reductions to reduce the number of board serials. Some boards with different board serials have the same board positions after rotation or mirroring; these are called *isomorphic boards*. If we rotate the board in Fig. 4 clockwise 90 degrees, then it becomes the board in Fig. 5. Therefore, the board in Fig. 4 and the board in Fig. 5 are isomorphic. Because of the property of isomorphic boards, we need to save only one state, which can significantly reduce the number of states. Therefore, except for the board with the lowest board serial among isomorphic boards, we consider the other board serials illegal.

Although we have succeeded in reducing the number of legal states, the scattered legal states are inefficient to store. Therefore, we use RRR^26^ to renumber these legal states. RRR is a data structure that can compress sparse bit arrays to store the same data in less space. RRR provides three operations, namely, $\textbf{access}$, $\textbf{rank}$, and $\textbf{select}$. $\textbf{Access}$ is an operation that obtains the bit value of an index. $\textbf{Rank}$ is an operation that returns the number of 1-bits before an index, and $\textbf{select}$ is an operation that returns the position of the kth 1-bit. $\textbf{Select}$ is the inverse of $\textbf{rank}$, which means that we have the following relationships.

2 $$\begin{aligned} \textbf{rank}(\textbf{select}(i)) + 1&= i \end{aligned}$$

3 $$\begin{aligned} \textbf{select}(\textbf{rank}(i) + 1)&= i \end{aligned}$$

For each board status, we perform RRR on the bit array representing whether the state is legal and use $\textbf{rank}$ to calculate the new serial of the states, named the *RRR serial*. Thus, using RRR serials, we can store all the endgame values in a continuous space. On the other hand, storing the children of all states is costly in storage. After observing the relationship between board statuses and actions, we propose an approach to share children with other board statuses. The details for sharing children are given in Supplementary Information SII.

### Verification

After building the endgame databases, we verify the correctness of these databases. We use two approaches to verify the endgame databases: consistency checking and checking previous publication data.

#### Consistency checking

For each state in the endgame databases, the player always chooses the best ply for him- or herself. For example, in $5\times 5$ Go, if there is one ply that leads the player to win-25, the endgame value of at least one child must also be win-25. Therefore, according to the above rule, we verify the consistency of all the states.

#### Checking previous publication data

In 2018, Zhang Xu published a problem set for $4\times 4$ Go^5^. Fukui Masaaki also published problem sets for $5\times 5$ Go^27^^28^ in 2000 and 2002. We use the problems in these books to verify the correctness of the constructed endgame databases. Although the rules used in these problem sets are Japanese Rules and our endgame databases use AGA Rules, the answers are the same in most cases. Only 7 out of 225 problems have different results because of the different scoring rules. One such example will be discussed in the following section. All 7 examples are given in Supplementary Information SIII.

### Level of difficulty

There is no explicit definition of problem difficulty in Go. However, players generally agree that certain positions are more challenging than others. Endgame databases can assist in determining the ply that will result in the highest game score for any given position. With this information, we aim to design a formula to quantify the difficulty or easiness of Go.

**Figure 6:**
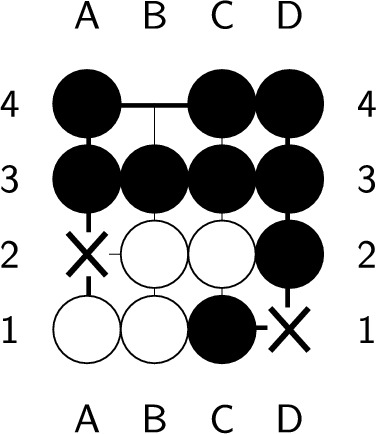
An example of an optimal ply. In the figure, ’X’ indicates the optimal ply.

**Figure 7:**
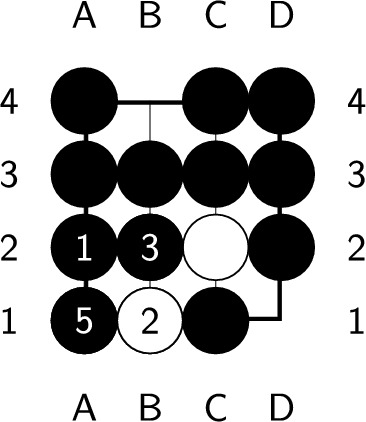
Optimal solution with a length of 5.

**Figure 8:**
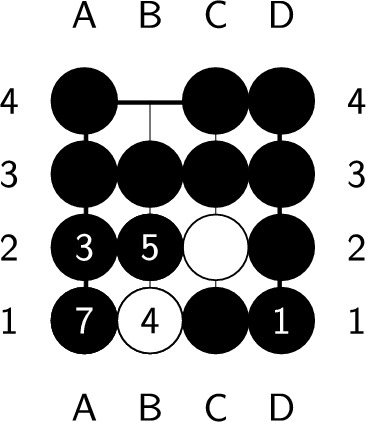
Optimal solution with a length of 7.

If the endgame value remains the same after playing a ply, it is considered optimal. However, if the endgame value decreases, which means you’ve lost a chance to get more scores, this ply is considered nonoptimal. An endgame value denotes the upper bound of the position’s score. Whatever the ply you play, the best score you can get is the endgame value. During constructing the endgame, we make sure there is at least one ply whose endgame value stays the same after it is played. Therefore, no ply can increase the endgame value, and a ply is optimal if and only if the endgame value remains the same after playing the ply. In Fig. 6, the black player plays next. Both a2 and d1 are optimal plies, but playing at d1 requires additional plies to capture all the opponent stones, as shown in Figs. 7 and 8. On the other hand, playing at b4 causes all of our stones to be captured, which results in lose-16. Therefore, b4 is not an optimal ply. Additionally, if we choose to pass, we cannot capture all of the white stones, which is also not an optimal ply.

#### Designing a formula for the easiness of positions

For a position, the legal moves can be divided into two categories depending on whether the move is optimal. Let *b* be a position with *N*(*b*) legal moves, $m_1, m_2,\dots , m_{N(b)}$, where the first *T*(*b*) are optimal. Let $s_i$ be the endgame value of $m_i$. We note the following observations that may affect the *easiness* of a position. Note that *opt*(*b*) is the optimal value for *b*.

***Ratio of optimal moves*** Intuitively, the greater the ratio of optimal moves among all legal moves is, the greater the chance of choosing one such move. Hence, we first note the following formula for the easiness of *b*.

4 $$\begin{aligned} E_{1}(b) = \frac{T(b)}{N(b)} \end{aligned}$$

***Distinguishability*** In addition to considering the chance of choosing optimal moves, it should be noted that when the values of nonoptimal moves are closer to the optimal value, human players may be easily confused and choose an incorrect move. Hence, in Eq. (5), we add weights to such nonoptimal moves and redefine easiness in Eq. (6). The weight is calculated by the difference between *opt*(*b*) and $s_i$.

5 $$\begin{aligned}{} & {} F(b): T(b) + \sum _{i=T(b)+1}^{N(b)}{\frac{c_{1}}{\exp {(\frac{|opt(b)-s_i|}{c_{2}})}}} \end{aligned}$$

6 $$\begin{aligned}{} & {} E_{2}(b) = \frac{T(b)}{F(b)} \end{aligned}$$

The weighting constants $c_1$ and $c_2$ determine the balance between the number of optimal moves and distinguishability. The value *c*1 denotes the upper bound of the weight that a nonoptimal ply can affect the difficulty. As a result, *c*1 is non-negative. On the other hand, when the difference between the endgame values of the nonoptimal plies and the optimal value becomes larger, *c*2 controls the magnitude of the decline in the influence. Thus, *c*2 can be any positive number greater than 1 because we need to ensure the influence falls as the differences become larger. Note that *c*1 and *c*2 need to be considered together. When $c_1$ and $c_2$ are large, *F*(*b*) is dominated by distinguishability. Based on the above properties, we find that the constants $c_{1} = 1.4$ and $c_{2} = 6$ best fit our intuition.

***The value of*** *opt*(*b*) If *opt*(*b*) is win-25 or lose-25, which according to Fig. 10 are common scenarios in Go, the game should be relatively easy since one side has a great advantage. Positions whose optimal values are closer to a draw are often difficult since both players have counter moves in a sequence of plies to follow. Hence, we use a hyperbolic equation, Eq. (7), to capture this idea.

7 $$\begin{aligned} G(b): \sqrt{\left( c_{3}\cdot opt(b)\right) ^{2}+1}-1 \end{aligned}$$

We set $c_{3} = \frac{\sqrt{3}}{25}$ to ensure the output range of *G*(*b*) is between 0 and 1. Finally, the easiness of *b* is given by Eq. (8).

8 $$\begin{aligned} E_{3}(b) = \frac{T(b)\cdot G(b)}{F(b)} \end{aligned}$$

## Experimental results

We next construct the $N \times N$ Go endgame databases for $N=2$ to 5.

### Experimental design

We used the specifications in Tables 4 and 5 to construct the endgames. The programs used were written in C++ and compiled with C++17 and O3 flags. Additionally, we used OpenMP to speed up the execution.

**Table 4:** The experimental settings for $2\times 2$ Go, $3\times 3$ Go, and $4\times 4$ Go were used.

CPU	Intel(R) Xeon(R) CPU E5-4650 0 @ 2.70GHz
Memory	192 GB
OS	FreeBSD 11.2-RELEASE-p14
Compiler	gcc 7.5.0
Parallel	OpenMP 3.1

**Table 5:** Experimental settings for $5\times 5$ Go.

CPU	Intel(R) Xeon(R) CPU E5-2699 v4 @ 2.20GHz
Memory	756 GB
OS	Ubuntu 16.04.6 LTS
Compiler	gcc 5.5.0
Parallel	OpenMP 4.0

We performed retrograde analysis for different sizes of Go, and the numbers of states are shown in Table 6. We also recorded the number of epochs and the duration of the retrograde analysis, as shown in Table 7. An epoch is an iteration in which all states are updated. As the size of the board increases, the number of epochs required also increases. With $5\times 5$ Go, as mentioned before, we spent much time loading files because we performed the retrograde analysis part by part.

**Table 6:** Numbers of states before dealing with cycles.

Size	Win	Draw	Lose	Unstable	Total
$2\times 2$	60	13	26	58	157
$3\times 3$	23,672	2,069	12,254	656	38,651
$4\times 4$	44,840,522	3,804,952	26,520,321	880,806	76,046,601
$5\times 5$	96,426,010,559	5,732,658,717	60,764,063,490	742,542,104	163,665,274,870

**Table 7:** Numbers of epochs used in the retrograde analysis.

Size	Number of epochs	Time spent (ms)
$2\times 2$	3	5
$3\times 3$	18	22
$4\times 4$	29	16,597
$5\times 5$	39	710,488,652

After removing the stable states, we found all the SCCs, including the in-cycle SCCs and the out-cycle SCCs. Table 8 shows the maximum and minimum values of the sizes of the in-cycle SCCs. In particular, there is only one in-cycle SCC for $2\times 2$ Go, but this SCC has more states than the other SCCs for $3\times 3$ Go and $4\times 4$ Go.

**Table 8:** Minimum/maximum sizes of SCCs.

Size	Minimum	Maximum
$2\times 2$	44	44
$3\times 3$	6	8
$4\times 4$	4	24
$5\times 5$	4	562

Finally, after dealing with the cycles, the results are shown in Table 9. For $2\times 2$ to $5\times 5$ Go, the number of winning states is greater than the number of losing states. Since we assume that all of the states are on the black player’s turn and that the results are recorded from the perspective of the black player, we believe that this is due to the advantage of having the first move. For $5\times 5$ Go, after using RRR, we reduced the space used by 99.36%.

**Table 9:** Numbers of states after dealing with cycles.

Size	Win	Draw	Lose	Unstable	Total
$2\times 2$	98	13	46	0	157
$3\times 3$	23,900	2,089	12,662	0	38,651
$4\times 4$	44,947,882	4,409,232	26,689,487	0	76,046,601
$5\times 5$	96,751,250,972	5,753,029,628	61,160,994,270	0	163,665,274,870

### Data analysis

**Figure 9:**
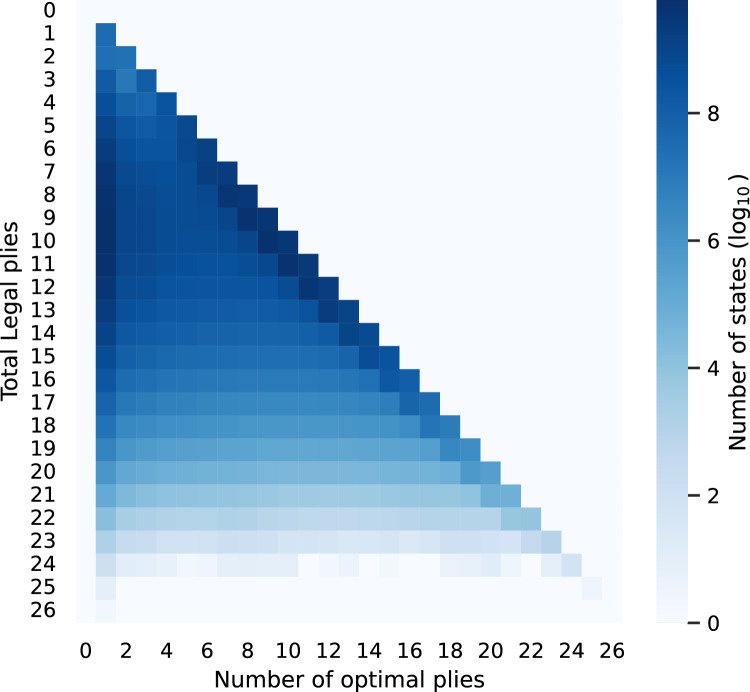
Plot of the numbers of states, using the $\log _{10}$ color scale on the right, under different number of optimal plies and legal plies.

We use $5\times 5$ Go to illustrate the data analysis. First, we calculate the number of best plies and the number of legal plies for each state. The values on a $\log _{10}$ color scale are shown in Fig. 9. There are only 15 or fewer legal plies in most of the states. In addition, many must-win and must-lose states appear on the diagonal line, which means that every ply leads to the same score. On the other hand, there are also many states with only one best ply, which means that many states have only one correct countermove.

**Figure 10:**
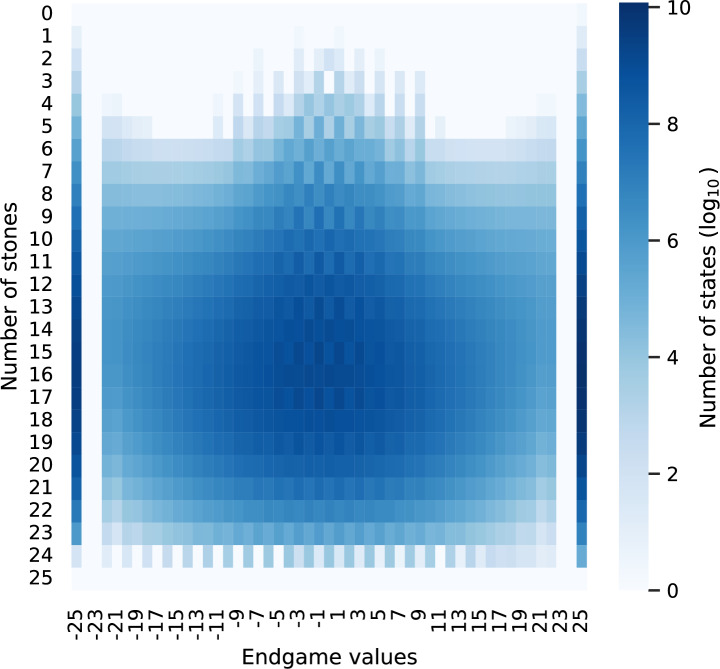
Plot of the numbers of states, using the $\log _{10}$ color scale on the right, under different endgame values and the number of stones.

Next, we perform statistical analysis based on the number of stones and the endgame values for all the states, as shown in Fig. 10. The values in Fig. 10 are on a $\log _{10}$ color scale. A negative endgame value means that the black player loses, and a zero endgame value means a draw. As shown in Fig. 10, most states are either win-25 or lose-25. In addition to win-25 and lose-25, there are many states whose endgame values are approximately draws. Furthermore, when more stones are placed on the board, we observe diverse endgame values.

### Results of initial states for different board sizes and rules used

To observe the effect of cycles, we construct two types of endgame databases with different rules. One is the AGA rule, called the No-Cycle endgame. The other allows cycles of lengths greater than 2, called the Cycle-Draw endgame. If the length of a cycle is greater than 2, the game ends with a cycle-draw. After building two different rule endgame databases for different sizes of Go, the endgame values of the initial states for different board sizes are as shown in Table 10. We found that allowing cycles gives the second player a chance to draw when the board size is even. When the board size is odd, the first player is sure to win regardless of whether cycles are allowed.

**Table 10:** Endgame values of the initial states for different board sizes.

Board size	No cycle	Cycle-draw
$2\times 2$	Win 1	Cycle-draw
$3\times 3$	Win 9	Win 9
$4\times 4$	Win 2	Cycle-draw
$5\times 5$	Win 25	Win 25

### Time taken by KataGo to solve problems of different difficulty levels

**Figure 11:**
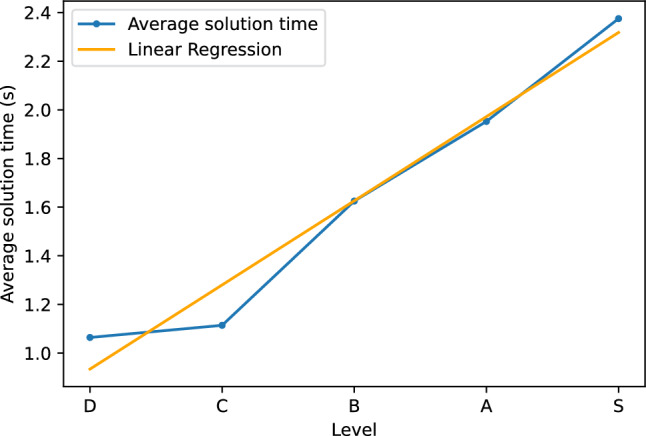
The average solution time for problems of each level.

KataGo is a state-of-the-art open-source deep learning Go engine that can handle various board sizes, including $4\times 4$ Go. We input $4\times 4$ Go problems from the book published by Zhang Xu^5^ into KataGo and recorded the time taken for KataGo to solve them. The problems are divided into five levels, S, A, B, C, and D, with S being the most difficult and D being the easiest. Figure 11 shows the average time taken by KataGo to solve problems of different levels. Due to the differences in Go rules between KataGo and the problems of Zhang Xu, we filtered out five level-A problems that can only be solved correctly for more than 17 seconds, whereas the rest 295 problems are solved within 9 seconds. We feel KataGo may have flaws in solving them. As shown, KataGo solves easier problems (levels B, C, and D) faster than more difficult problems (levels S and A). The correlation coefficient of the average solution time is 0.98. Therefore, the time taken by KataGo can be used as a measure of the difficulty of a problem.

Although KataGo’s solution time can indicate the difficulty of a problem, the reasons making some positions spend more time than others are not known. Additionally, KataGo takes time to judge the difficulty of a problem, which is unacceptable when it is needed to examine and compare a huge number of positions. Therefore, we aim to develop a formula to address the difficulty measure that KataGo provides, with the aim of giving Go players insights into why some positions are more difficult than others.

### Correlation between $E_{3}(b)$ and the time KataGo needs to solve *b*

**Figure 12:**
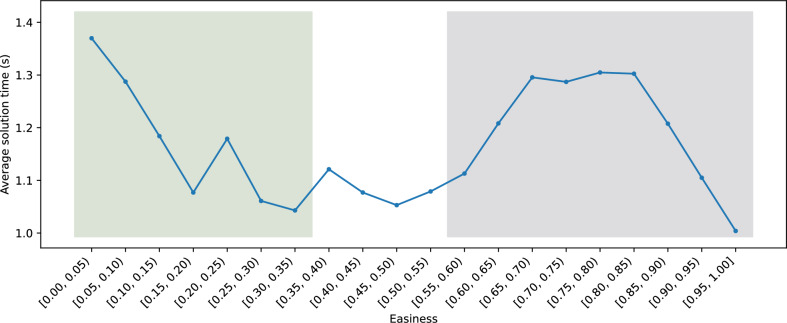
The average solution time for each group.

Equation (8) outputs a value between 0.0 and 1.0 and is used to capture the easiness of a position in Go. We divided the states into groups according to their $E_{3}(b)$ values. Each group had a range of 0.05, with 1.0 being the last group. Then, we randomly selected 1000 games in each group to be tested by KataGo. For each game, we recorded the number of seconds required for KataGo to find the correct answer. In the end, we averaged the time required for each group of games. The results are shown in Fig. 12.

In Fig. 12, on average, when $E_{3}(b)$ is between 0.0 and 0.05, the games take the longest time to solve; conversely, the shortest time is taken when $E_{3}(b)$ is between 0.95 and 1.0. We find that when $E_{3}(b) \le 0.35$, our formula has a strong correlation with the average time KataGo needs to solve *b*. When $E_{3}(b)$ is above 0.35, and especially when it is above 0.6, the formula is not accurate.

**Figure 13:**
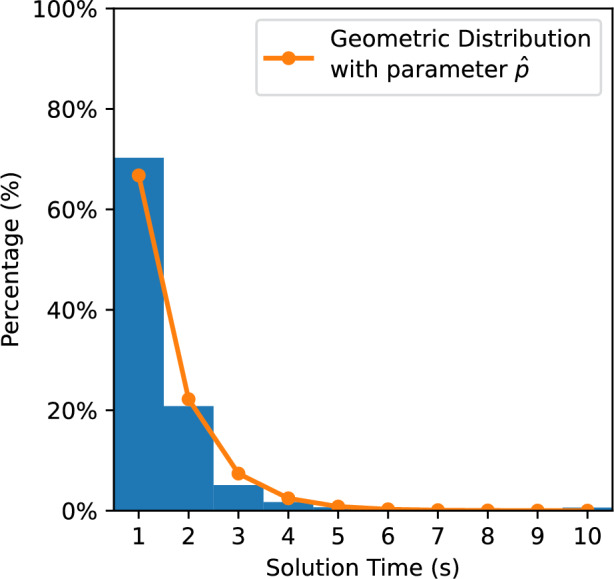
Histogram of the solution time needed versus the percentage of positions solved when $E_{3}(b) \in [0.0, 0.02)$.

**Figure 14:**
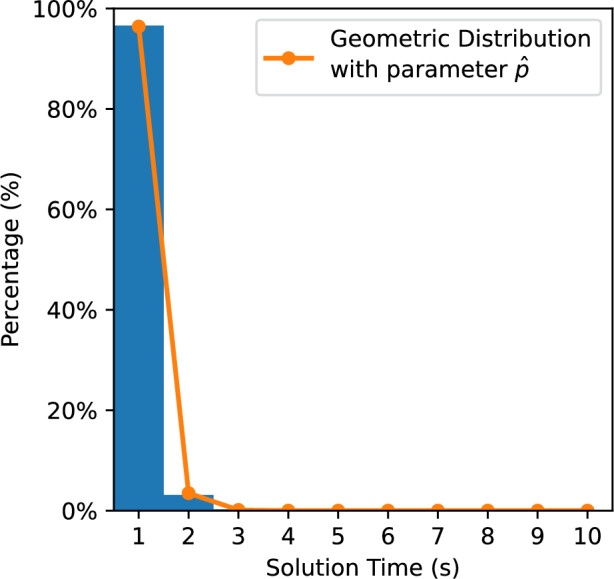
Histogram of the solution time needed versus the percentage of positions solved when $E_{3}(b) \in [0.34, 0.36]$.

**Figure 15:**
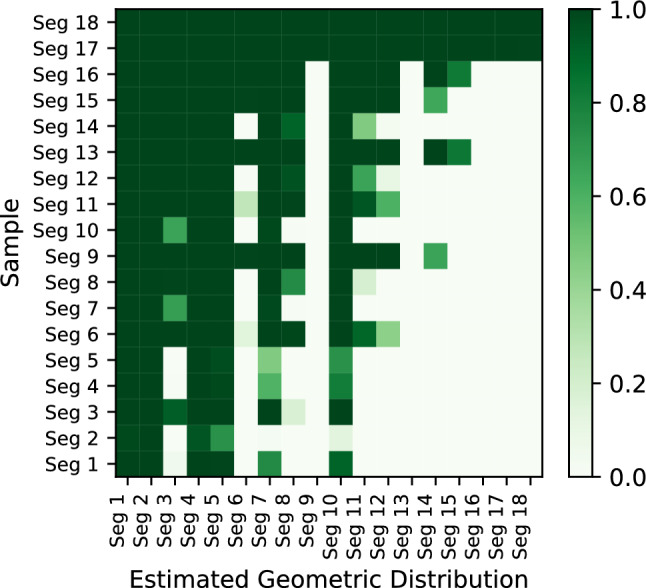
The p values of the chi-square goodness of fit test.

We performed additional tests on the cases between 0 and 0.36. We further subdivided these cases into 18 segments. Each segment has a range of 0.02. Similarly, we input the games into KataGo and recorded the solution times, with 10,000 randomly selected positions for each segment. Figures 13 and 14 show the histograms of the solution time needed versus the percentage of positions solved for Segment 1 and Segment 18, respectively. Figures 13 and 14 show that the results have a geometric distribution with a success probability *p*. Therefore, we tested whether the samples follow a geometric distribution by using the chi-square goodness of fit test. Figure 15 shows the *p* values of the tests. We then checked whether the data in a segment fit a geometric distribution with a success probability ${\hat{p}}$ by estimating ${\hat{p}}$ using maximum likelihood estimation (MLE). We found that the sample data of most segments fit. In addition, according to the figure, there is high confidence that Segment 1 and Segment 18 do not come from the same geometric distribution.

## Discussion

### Differences in Go rules

**Figure 16:**
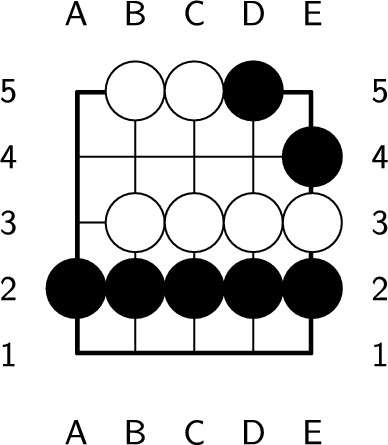
A sample position whose answer deviates from the published results.

**Figure 17:**
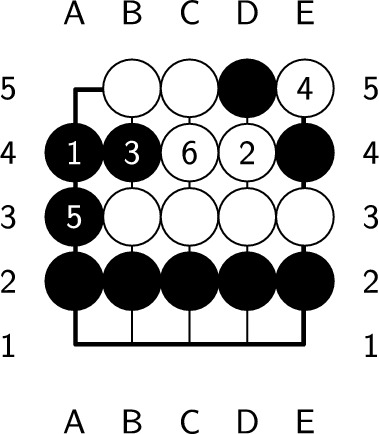
Answer for the board in Fig. 16 using Japanese Rules.

**Figure 18:**
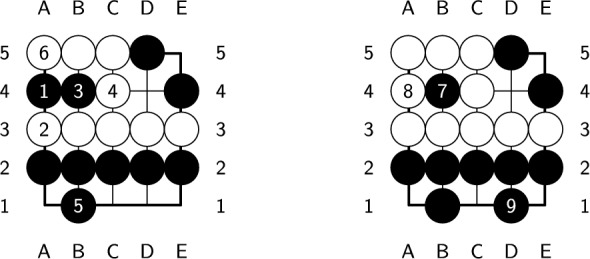
Answer for the board in Fig. 16 using AGA Rules.

In the previous section, we verified the correctness of the constructed endgame databases by using the problems in a published book^28^. Figure 16 shows one of the 7 examples found to deviate from the answer given in the book^28^. The answer is shown in Fig. 17. Since the white player captures two black stones in the fourth ply and three fewer empty intersections than the black player, the white player ultimately obtains lose-1 using the Japanese Rules. The white player also obtains lose-1 under the AGA Rules. However, captured stones are not counted in the score according to the AGA Rules. If we play the same plies as in Fig. 17, then the black player ultimately scores three more points than the white player does, which results in the white player obtaining lose-3. The optimal moves according to the AGA Rules are shown in Fig. 18. The white player should play at a3 instead of d4 at the second ply to obtain lose-1. This example shows the difference between using different rules. All 7 examples are shown in Supplementary Information SIII.

### How well $E_{3}(b)$ measures the level of difficulty

#### Observations

**Figure 19:**
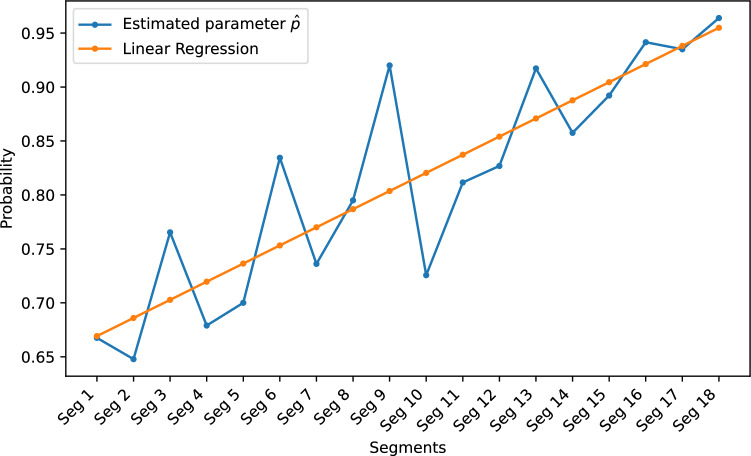
Plot of the estimated parameters ${\hat{p}}$ of all segments and a linear regression of ${\hat{p}}$.

**Figure 20:**
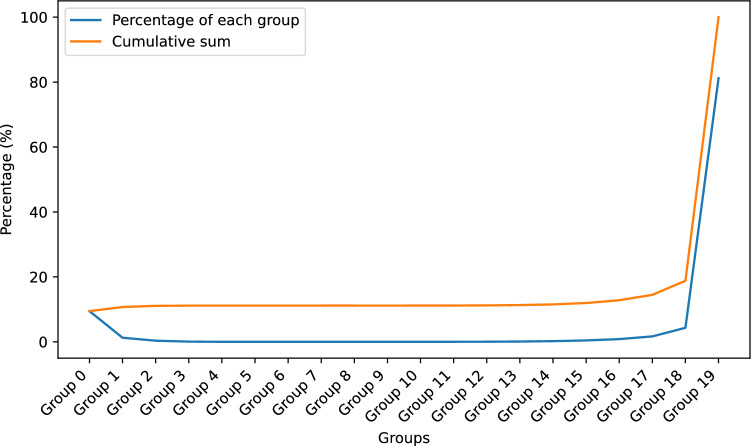
Percentages of positions within each group.

In the previous section, we subdivided the area between 0 and 0.36 into 18 segments. We assume KataGo builds a model that has a probability *p* of solving game *b* depending on how easy *b* is every second it runs. We thus fit the geometric distributions of all 18 segments to determine the changes in probability *p*. The results are shown in Fig. 19. The R-squared ($R^2$) value of the fit is 0.758, which indicates good agreement. The estimated parameters ${\hat{p}}$ for Segment 1 and Segment 18 are 0.6676 and 0.9639, respectively, which shows that Segment 1 is more difficult than Segment 18 is. In addition, Fig. 19 illustrates that the value of ${\hat{p}}$ increases as the value of easiness increases, which means that Eq. (8) works well when $E_{3}(b) \le 0.35$. We checked all $5\times 5$ Go positions and found that $11.2 \%$ of them were within this range. Figure 20 shows the percentage of positions within each group.

#### Limitations

**Figure 21:**
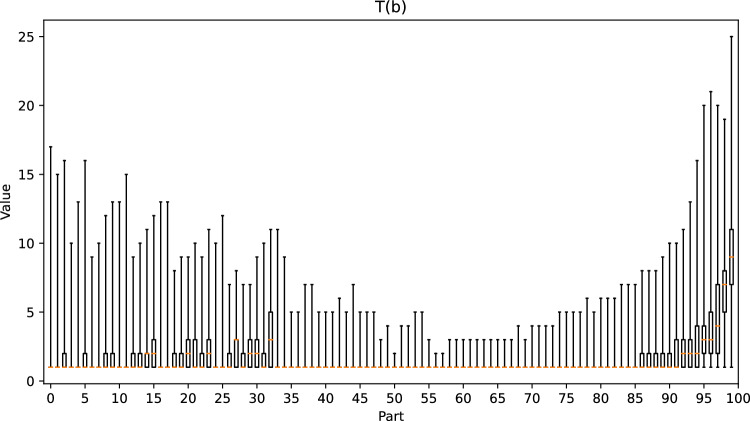
Box plot of *T*(*b*) for each part.

**Figure 22:**
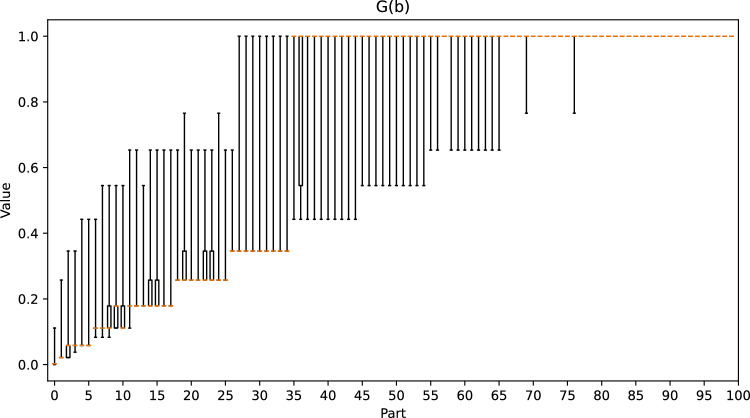
Box plot of *G*(*b*) for each part.

**Figure 23:**
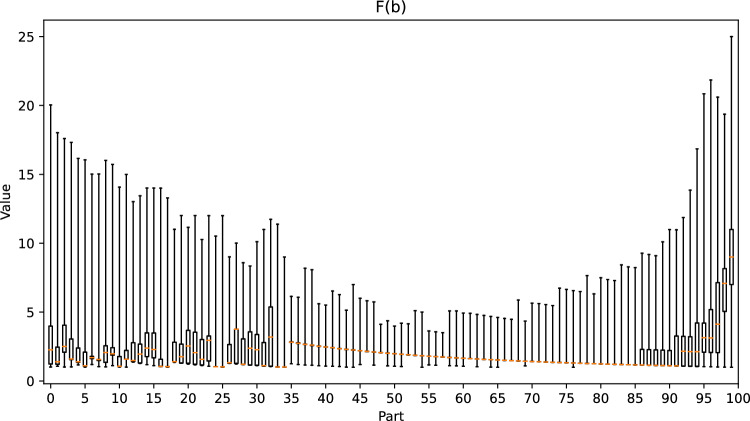
Box plot of *F*(*b*) for each part.

Figure 12 shows that our method works well when $E_{3}(b) \le 0.35$. To test the correctness of our observations, we divided the positions into 101 parts according to easiness, and we present the values of *T*(*b*), *G*(*b*), and *F*(*b*) for each part in the box plots in Figs. 21, 22 and 23, respectively. When $E_{3}(b) \le 0.35$, we observe that as the value of easiness increases, the values of *T*(*b*) and *G*(*b*) both increase, and the maximum value of *F*(*b*) decreases, which corresponds to our observations. However, when $E_{3}(b) > 0.35$, *T*(*b*) and *G*(*b*) are generally close to 1.0, which results in a negative correlation between $E_{3}(b)$ and *F*(*b*). Therefore, in addition to our observations, domain-specific knowledge is required to determine the optimal ply that is not currently included in the formula. In a life-death problem, there is usually only one optimal move, and only one side can survive. If one makes a mistake, one is likely to lose all territory. Hence, $T(b) = 1$ and $opt(b) = 25$, which imply a high *G*(*b*) and a low *F*(*b*). Although this position is difficult, we still obtain a high score from Eq. (8).

**Figure 24:**
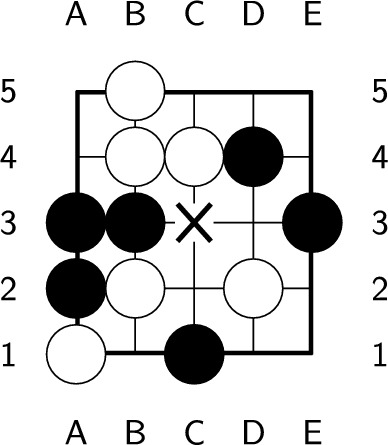
An example of a difficult position that has a high $E_{3}(b)$ value, 0.938. In the figure, ’X’ indicates the optimal ply.

**Figure 25:**
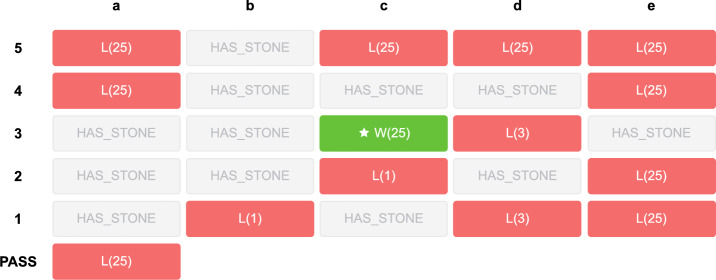
Endgame values after playing each legal move of the board in Fig. 24.

Taking the board in Fig. 24 as an example, it is the black player’s turn, and the optimal ply is c3. Figure 25 shows the endgame values after each legal move. If the black player plays at c3, the white stones can be captured since there is no space for the white player to form two eyes to prevent capture. Finally, the black player obtains win-25. On the other hand, if the black player makes other moves, the white player can play at c3, and the white stones survive. In the end, the black player loses since the white player can occupy the most territory. This example is relatively difficult since it requires specialist knowledge to find the exact answer. However, $E_{3}(b)$ reaches 0.938, where $T(b) = 1.0$, $G(b) = 0.999$, and $F(b) = 1.066$. The value of easiness is obviously not correct. Thus, without considering specialist knowledge, the easiness of positions may lead to mistakes.

## Concluding remarks and future work

In Go, cycles can occur during the game due to a special rule: capturing. However, in some rules, cycles are forbidden, which makes it almost impossible to use retrograde analysis directly when building endgame databases. In this paper, we provide an approach for building endgame databases without cycles. With SCCs, we can handle cycles and determine the endgame values of cycles according to the rules used. We also use RRR to reduce the memory usage when working with larger board sizes. After building endgame databases using different rules and different board sizes, we observe that the second player has a chance to draw with the first player only when the board size is even and cycles are allowed. Finally, we make several observations regarding why some positions are easier than others, which enables us to easily identify positions with a given level of difficulty with a high probability. This formula can be used in computerized tutoring systems to help humans improve their Go playing skills.

Although we are able to determine easier positions with a high probability, we cannot easily recognize more difficult ones perhaps due to no specialist knowledge being used. The KataGo solution times of 5 out of 300 positions from the expert’s annotated book^5^ are much more than the average solution time of the rest 295 ones as mentioned in Sect. "Time taken by KataGo to solve problems of different difficulty levels". All of them are level-A, and it appears that more insights into those positions are needed to remedy possible shortcomings of KataGo. This will be the focus of future work.

### Supplementary Information

Supplementary Information.

## Data Availability

The analysis data in this study are available from the corresponding author upon reasonable request.
