# Supplementary material for: Construction, observation and knowledge abstraction for go endgames on small boards
Source: Sci Rep. 2024 Mar 22;14:6903. doi: 10.1038/s41598-024-57338-x (PMC10959957; doi:10.1038/s41598-024-57338-x)
Supplement: Supplementary file 1 — Supplementary Information. [file 41598_2024_57338_MOESM1_ESM.pdf]

Construction, Observation and Knowledge Abstraction for small-board-size Go Endgame
Supplementary Information

Chia-Ming Hsu[1], Hung-Cheng Lin[2], Yueh-Ting Chen[2], Chih-Wen Hsueh[2], and Tsan-sheng Hsu[1,*]

[1]Institute of Information Science, Academia Sinica, Taipei, 115201, Taiwan
[2]Department of Computer Science and Information Engineering, National Taiwan University, Taipei, 106216, Taiwan
[*]tshsu@iis.sinica.edu.tw

SI Data Structure and Implementation

Board serial and Status code

A state consists of a board position, a board status, and a turn. To reduce the number of states, we assume that all states are always the black player's turn. If a state is the white player's turn, then we swap the black and white stones. The black player in the swapped state is as same as the white player in the original state. Therefore, we save the cost of recording the turn. We use numbers to represent the board positions and the board statuses, called *board serial* and *status code* respectively. Finally, we combine the two numbers to form a state index.

Board serial

The board serial is an integer to encode the board position. To give each board position a number, we need to find a function that takes a board position and outputs a number. For convenience, the function must be injective. Finally, we choose the ternary number system to construct the function, because each intersection may be a black stone, a white stone, or empty. Taking 4x4 Go as an example, Equation S1 shows the function for computing the board serial of a position. The number i represents the serials of the intersection as shown in Table S1. The number b_i represents the condition of intersection i, as listed in Equation S2.

$$Serial_B = \sum_{i=0}^{15} b_i \times 3^i \qquad \text{(S1)}$$

$$b_i = \begin{cases} 0 & \text{Intersection } i \text{ is empty} \\ 1 & \text{Intersection } i \text{ is a black stone} \\ 2 & \text{Intersection } i \text{ is a white stone} \end{cases} \qquad \text{(S2)}$$

0	1	2	3
4	5	6	7
8	9	10	11
12	13	14	15

Table S1: Serial of intersection in 4x4 Go.

Status code

In the same board position, there may or may not be a ko on the board, depending on the game history. To distinguish between these differences, we use the board status to record the game history. There are 7 types of board status, KO, NO-KO-NO-PASS, NO-KO-PASS-1, NO-KO-PASS-2, NO-KO-PASS-1-KO, NO-KO-PASS-2-KO, and INITIAL. Table S2 is a detailed introduction to each board status. The beginning state, INITIAL, is a special state denoting the initial board. On the other hand, since a KO state becomes a state without KO after two passes, it needs two more passes to end the game. To distinguish whether starting from a state with KO or starting from a state without KO, the states after passing once and twice from the state with KO are NO-KO-PASS-1-KO and NO-KO-PASS-2-KO respectively.

Status	Introduction
KO	There is a ko on the board.
NO-KO-NO-PASS	No player has passed nor any ko on the board.
NO-KO-PASS-1	A player has passed.
NO-KO-PASS-2	Both players have passed.
NO-KO-PASS-1-KO	A player has passed after a ko on the board.
NO-KO-PASS-2-KO	Both players have passed after a ko on the board.
INITIAL	The beginning of the game.

Table S2: Board status.

For convenience, each board status is assigned with a number, called the *status code*. KO status needs to record the position of the ko. Thus, N x N Go has $N \cdot N + 6$ different status codes. Table S3 shows all the status codes and the status code of KO status uses the serial of intersection, as shown in Table S1, to represent the ko position.

Board status	Status Code
KO	ko position
NO-KO-NO-PASS	10000_2
NO-KO-PASS-1	10001_2
NO-KO-PASS-2	10010_2
NO-KO-PASS-1-KO	10011_2
NO-KO-PASS-2-KO	10100_2
INITIAL	10101_2

Table S3: Status code in 4x4 Go.

Illegal state index

We use 32 bits to represent a state index in 4x4 Go, but some state indices are not legal. In Go rules, the stones with no liberty must be removed from the board. Therefore, we need to label these serials as illegal. In addition, for some board statuses, there are some unreasonable board positions that should not exist. For example, a board position with no stone is illegal when the board status is NO_KO_NO_PASS. Table S4 shows all the rules we use to check if the board position is legal in a particular board status.

Illegal conditions
There is a stone on the board when the board status is INITIAL.
There are stones without liberty on the board.
No white stones on the board, when the board status is KO, NO-KO-NO-PASS, NO-KO-PASS-1-KO and NO-KO-PASS-2-KO.
When the board status is KO at k, but k cannot be able to form a ko.
When the board status is NO-KO-PASS-1-KO or NO-KO-PASS-2-KO, no ko can form anywhere on the board.

Table S4: Illegal Conditions

Positions

We calculate and save the child indices of each state. According to the rules of Go, a player has only two choices: a move or a pass, and the maximum number of locations a player can play on a board is no more than the board size. So a state can have at most $N \times N + 1 = N^2 + 1$ children in N x N Go, which results in the game tree of Go being sparse. To store a sparse graph, we use the compressed sparse row (CSR) format as following to store the adjacency matrix of the game tree, instead of storing a two-dimensional array. We store two arrays: location array and children array. The children of each state are placed in consecutive positions in the children array. The location array records the beginning position of each state's children in the children array.

SII Child Positions Sharing across Different Board Statuses

For the states with the same board serial and different board statuses, the children of states are mostly the same, except for when ko happens. If the board status is KO, it has one less child. The other difference is with regard to pass. The board status of the child state after a pass depends on the board status of the current state, as shown in Table S5. In Table S5, the only difference is when the action is pass. Therefore, after dealing with the above differences, the states with the same board serial and different board statuses can share the same children, which can significantly reduce the storage space required.

Initial \ Ply	Capture (ko)	Move	Capture	Pass
KO	KO	NO-KO-NO-PASS	NO-KO-NO-PASS	NO-KO-PASS-1-KO
NO-KO-NO-PASS	KO	NO-KO-NO-PASS	NO-KO-NO-PASS	NO-KO-PASS-1
NO-KO-PASS-1	KO	NO-KO-NO-PASS	NO-KO-NO-PASS	NO-KO-PASS-2
NO-KO-PASS-2	--	--	--	--
NO-KO-PASS-1-KO	KO	NO-KO-NO-PASS	NO-KO-NO-PASS	NO-KO-PASS-2-KO
NO-KO-PASS-2-KO	KO	NO-KO-NO-PASS	NO-KO-NO-PASS	NO-KO-PASS-1
INITIAL	--	NO-KO-NO-PASS	--	NO-KO-PASS-1

Table S5: Changing of board status after a ply is played.

To deal with ko, we store 25 bits to represent whether the player can play at the intersection. Also, according to the order of each legal move in the 25 bits, we place the children in the array in order. When a move becomes illegal due to a ko, we can use the 25 bits to locate and remove the corresponding child. On the other hand, to deal with the pass, we store the board status and the RRR serial separately. Then, we can modify the board status to any value desired. However, since the legal states in different board statuses are not the same, the two states could have different RRR serials even though the two states have the same board serial. To solve this problem, we align the RRR serials. For those board serials, we insert a **Null** value into the array, then the same extended RRR serials represent the same board serial in different board statuses.

However, if we insert too many **Null** values, it takes up too much space. So we need to choose the appropriate board statuses to share the children. The relationship of legal states in all board statuses is as Equation S3. Finally, we divide the board statuses into three groups, as shown in Table S6. However, in group 3, the RRR serials of the states are different after pass moves. Therefore, we store two states for the pass move.

$$\begin{gathered} \begin{matrix}\text{NO-KO-PASS-1-KO}\\ \text{NO-KO-PASS-2-KO}\end{matrix} \subset \text{NO-KO-NO-PASS} \subset \begin{matrix}\text{NO-KO-PASS-1}\\ \text{NO-KO-PASS-2}\end{matrix} \\ \text{NO-KO-PASS-1} = \text{NO-KO-PASS-2} \\ \text{KO} \subset \text{NO-KO-PASS-2-KO} \end{gathered} \tag{S3}$$

Group 1	NO-KO-NO-PASS, NO-KO-PASS-1
Group 2	NO-KO-PASS-1-KO
Group 3	NO-KO-PASS-2-KO, KO

Table S6: Groups of the shared children.

SIII Deviations of Constructed Endgame and Published Results

Position 1

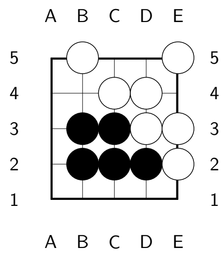

Figure S1: Position 1.

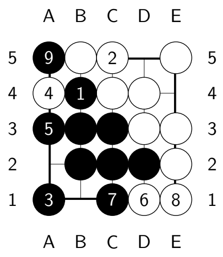

Figure S2: Answer of Position 1 using Japanese Rules.

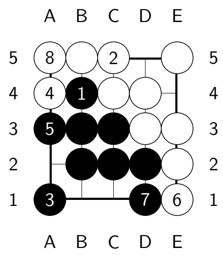

Figure S3: Answer of Position 1 using AGA Rules.

Position 2

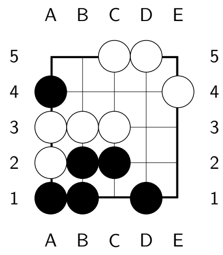

Figure S4: Position 2.

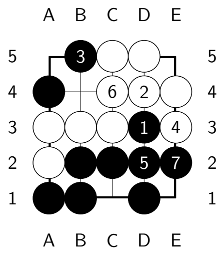

Figure S5: Answer of Position 2 using Japanese Rules.

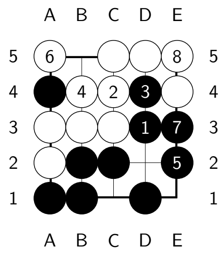

Figure S6: Answer of Position 2 using AGA Rules.

Position 3

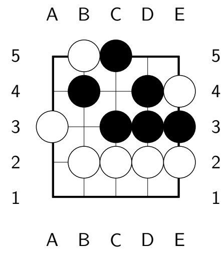

Figure S7: Position 3.

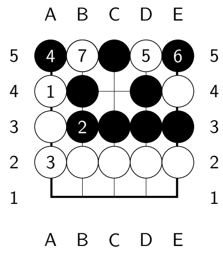

Figure S8: Answer of Position 3 using Japanese Rules.

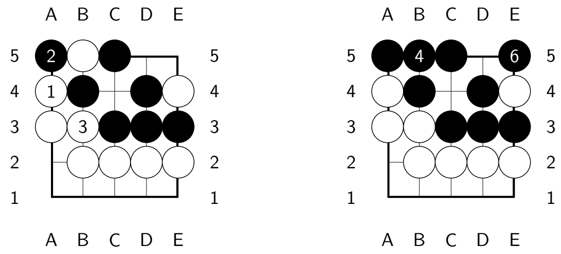

Figure S9: Answer of Position 3 using AGA Rules.

Position 4

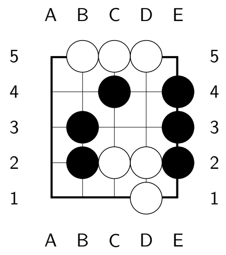

Figure S10: Position 4.

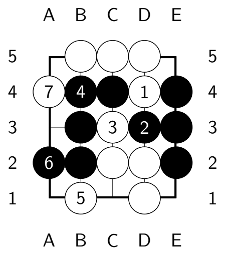

Figure S11: Answer of Position 4 using Japanese Rules.

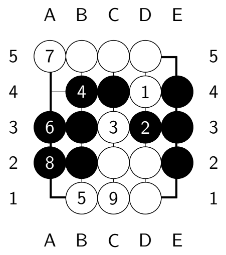

Figure S12: Answer of Position 4 using AGA Rules.

Position 5

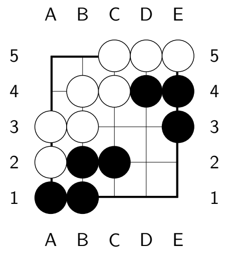

Figure S13: Position 5.

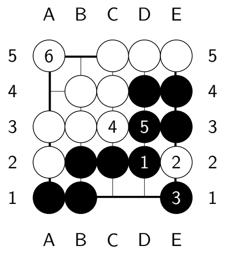

Figure S14: Answer of Position 5 using Japanese Rules.

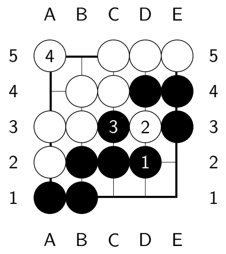

Figure S15: Answer of Position 5 using AGA Rules.

Position 6

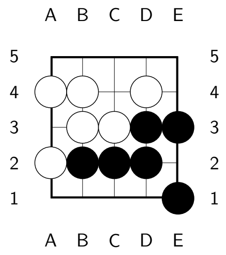

Figure S16: Position 6.

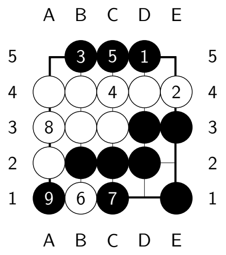

Figure S17: Answer of Position 6 using Japanese Rules.

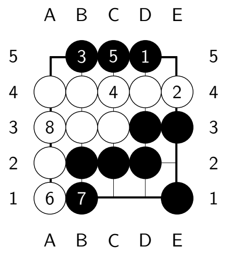

Figure S18: Answer of Position 6 using AGA Rules.

Position 7

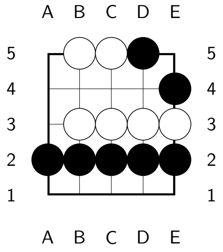

Figure S19: Position 7.

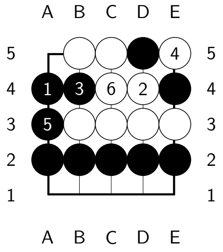

Figure S20: Answer of Position 7 using Japanese Rules.

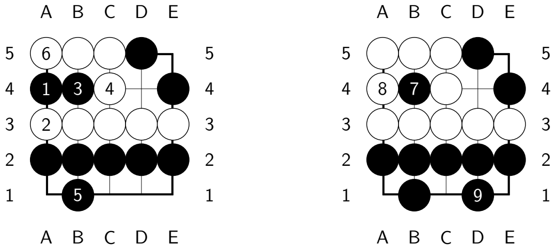

Figure S21: Answer of Position 7 using AGA Rules.

SIV Figure legends

S1 Position 1.

S2 Answer of Position 1 using Japanese Rules.

S3 Answer of Position 1 using AGA Rules.

S4 Position 2.

S5 Answer of Position 2 using Japanese Rules.

S6 Answer of Position 2 using AGA Rules.

S7 Position 3.

S8 Answer of Position 3 using Japanese Rules.

S9 Answer of Position 3 using AGA Rules.

S10 Position 4.

S11 Answer of Position 4 using Japanese Rules.

S12 Answer of Position 4 using AGA Rules.

S13 Position 5.

S14 Answer of Position 5 using Japanese Rules.

S15 Answer of Position 5 using AGA Rules.

S16 Position 6.

S17 Answer of Position 6 using Japanese Rules.

S18 Answer of Position 6 using AGA Rules.

S19 Position 7.

S20 Answer of Position 7 using Japanese Rules.

S21 Answer of Position 7 using AGA Rules.
